# “In the light of evolution:” keratins as exceptional tumor biomarkers

**DOI:** 10.7717/peerj.15099

**Published:** 2023-03-17

**Authors:** Işıl Takan, Gökhan Karakülah, Aikaterini Louka, Athanasia Pavlopoulou

**Affiliations:** 1 Izmir Biomedicine and Genome Center, Izmir, Turkey; 2 Izmir International Biomedicine and Genome Institute, Dokuz Eylül University, Izmir, Turkey; 3 DNA Damage Laboratory, Department of Physics, School of Applied Mathematical and Physical Sciences, National Technical University of Athens, Athens, Greece; 4 Section of Cell Biology and Biophysics, Department of Biology, School of Sciences, National and Kapodistrian University of Athens, Athens, Greece

**Keywords:** Cancer, Evolution, Comparative genomics, Phylogeny, Data mining, Interaction network, Gene expression patterns, Natural language processing

## Abstract

Keratins (KRTs) are the intermediate filament-forming proteins of epithelial cells, classified, according to their physicochemical properties, into “soft” and “hard” keratins. They have a key role in several aspects of cancer pathophysiology, including cancer cell invasion and metastasis, and several members of the KRT family serve as diagnostic or prognostic markers. The human genome contains both, functional *KRT* genes and non-functional *KRT* pseudogenes, arranged in two uninterrupted clusters on chromosomes 12 and 17. This characteristic renders KRTs ideal for evolutionary studies. Herein, comprehensive phylogenetic analyses of KRT homologous proteins in the genomes of major taxonomic divisions were performed, so as to fill a gap in knowledge regarding the functional implications of keratins in cancer biology among tumor-bearing species. The differential expression profiles of *KRTs* in diverse types of cancers were investigated by analyzing high-throughput data, as well. Several *KRT* genes, including the phylogenetically conserved ones, were found to be deregulated across several cancer types and to participate in a common protein-protein interaction network. This indicates that, at least in cancer-bearing species, these genes might have been under similar evolutionary pressure, perhaps to support the same important function(s). In addition, semantic relations between KRTs and cancer were detected through extensive text mining. Therefore, by applying an integrative *in silico* pipeline, the evolutionary history of KRTs was reconstructed in the context of cancer, and the potential of using non-mammalian species as model organisms in functional studies on human cancer-associated *KRT* genes was uncovered.

## Introduction

Cancer is a leading cause of death, currently accounting for one in six deaths worldwide (World Health Organization, https://www.who.int/news-room/fact-sheets/detail/cancer). It is a group of diseases characterized by abnormal cell growth, potentially invading neighboring tissues and/or spreading to other part(s) of the body (World Health Organization, https://www.who.int/health-topics/cancer). It is denoted by diversities, complexities and, often, unpredicted dynamics in disease progression or metastasis (Williams, 2015; Williams, Zaidi & Sengupta, 2022). On the other hand, recent discoveries of earliest hominin malignancies dating millions of years make paleontology, epidemiology, history, systems, and phylogenetic analyses feasible for elucidating underexplored underlying mechanisms and cancer patterns (Williams, 2015; Faltas, 2011; Wahba, Herrerín & Sánchez, 2021; Dittmar et al., 2020).

Current anticancer modalities include radiation, chemotherapy, and surgery. Nonetheless, DNA damage and genome instability caused by radiation and chemotherapy have also several adverse effects and in many cases cause toxicity. A percentage of patients may initially respond to cancer, but eventually develop resistance to therapy and disease recurrence, while current treatments may frequently result in a ‘tsunami’ that kills both cancer and healthy cells non-selectively, often leading to side-effects (Nikolaou et al., 2018; Toy et al., 2021; Pavlopoulou et al., 2017; Emran et al., 2022). A deeper understanding of the molecular determinants and mechanisms that govern carcinogenesis would likely enable the identification of reliable diagnostic and prognostic biomarkers, improvement of clinical decision-making as well as the improvement of targeted therapies.

The emerging field of evolutionary medicine or “Darwinian medicine” allows the investigation of human diseases from an evolutionary perspective (Nesse, 2001; Kranke, 2022; Logotheti et al., 2022). Thus, deciphering the genetic and molecular factors/mechanisms that contribute to shaping disease evolution, by examining their conservation across diverse species, would probably increase our knowledge regarding the etiology, development, progression and treatment of chronic diseases like cancer (Lineweaver, Davies & Vincent, 2014; Marquardt et al., 2021; Trigos et al., 2017). Multigene families like kallikreins (Pavlopoulou et al., 2010), cathelicidins (Kosciuczuk et al., 2012), *MAPK* (mitogen-activated protein kinases) (Li, Liu & Zhang, 2011), carcinoembryonic antigens (Pavlopoulou & Scorilas, 2014), *OXPHOS* (oxidative phosphorylation) (De Grassi, Lanave & Saccone, 2008) and *T2R* (bitter taste receptor) (Dong, Jones & Zhang, 2009), which have undergone a series of gene duplications, are implicated in critical pathophysiological processes. In this regard, herein, we aimed to investigate the role of the extended *keratin* gene family in cancer from an evolutionary point of view.

Keratins (KRTs) are intermediate-filament-forming proteins present in epithelial cells, which are broadly classified into “hard keratins’’ and ‘‘soft keratins’’, on the basis of their physicochemical properties and their sulfur content (Bragulla & Homberger, 2009; Schweizer et al., 2006; Zhang & Fan, 2021). Hard keratins make up morphological structures in birds (scales, claws and feathers) and mammals (hair and nails). Soft epithelial keratins are highly involved in epithelial cell protection from mechanical and non-mechanical stressors, and regulate a number of cellular processes, such as apical–basal plasma membrane polarity, cell size, cell motility, protein synthesis, membrane trafficking, wound healing, cell growth and cell death (Moll, Divo & Langbein, 2008; Coulombe & Omary, 2002; Sarma, 2022). The human genome encodes both functional keratin genes and non-functional keratin pseudogenes (KRT8/18/19P), which are located in two clusters on chromosomes 12 (type II keratins except KRT18) and 17 (type I keratins) (Bowden, 2005; Hesse et al., 2004; Jacob et al., 2018).

There is accumulating evidence that keratins are involved in critical aspects of cancer, including invasion and metastasis (Elazezy et al., 2021; Yu et al., 2022; Sharma et al., 2019). In particular, changes in the activity of *E-cadherin*, which plays a critical role in epithelial-mesenchymal transition (EMT), increased the expression of *vimentin*, a well-studied marker in EMT, as well as keratin 17 in skin squamous cell carcinoma (Lan et al., 2014). It has also been shown that the interaction between vimentin and keratin 14 is essential for epidermal cell migration in epithelial cells (Velez-delValle et al., 2016). Keratin 16 has recently been linked to cancer metastasis and EMT as well (Elazezy et al., 2021). Therefore, keratins are used as commercially available markers to diagnose cancer: TPS (KRT18), TPACYK (KRT8/18) and CYFRA 21-1 (KRT19) (Bodenmuller et al., 1994; Lane & Alexander, 1990; van Dalen, 1996). Keratins are also considered as prognostic indicators in several epithelial cancers and they are implicated in treatment responsiveness (Vaidya, Dmello & Mogre, 2022; Welch et al., 2019; Werner, Keller & Pantel, 2020).

In this study, *in silico* systems biology approaches were employed, including comparative genomics, phylogenetics, high-throughput data processing, network-based methods, and natural language processing, in order to enhance our understanding regarding the functional implications of keratins in cancer biology among tumor-bearing species.

## Materials and Methods

### Database searching

The approved symbols and names of *Homo sapiens* keratins were collected from the HUGO Gene Nomenclature Committee (HGNC) database (https://www.genenames.org/; (Tweedie et al., 2021)) (Tables S1 and S2).

### Retrieval of KRT protein sequences

The human *KRT* gene symbols were used to retrieve the corresponding KRT protein sequences from the sequence database NCBI’s RefSeq release 210 (O’Leary et al., 2016). Given that the protein sequences are more evolutionarily conserved compared to their corresponding nucleotide sequences, the proteins encoded by the *KRT* genes were used in the phylogenetic analyses.

### Cross-genome search for KRT orthologs

The prototypic human type I and type II KRT protein sequences were used to search the well-annotated genomes of the species *Mus musculus* (Mouse), *Bos taurus* (Cow), *Gallus gallus* (Chicken), *Xenopus tropicalis* (Frog) and *Danio rerio* (Zebrafish) for corresponding orthologs in the databases ENSEMBL release 105 (Cunningham et al., 2022), NCBI’s RefSeq release 210 (O’Leary et al., 2016) and UniProtKB release 2021_04 (The UniProt Consortium, 2019) in an iterative manner until no novel putative sequences could be detected, by employing reciprocal BLASTP and TBLASTN (Altschul et al., 1990). The Translate program (http://web.expasy.org/translate/) was utilized to translate any nucleotide sequences into amino acid sequences.

Regarding the nomenclature of the KRT sequences in mouse, cow, chicken, frog and zebrafish, they were named based on their homology to their closest related well-annotated human KRT gene. Those KRT homologs with no significant sequence similarity to the fellow human KRTs (*i.e*., forming separate branches), they were arbitrarily referred to as “orphan” KRTs.

### Phylogenetic inference

The corresponding KRT protein sequences were extracted from the relevant databases. Subsequently, alignment of the full-length KRT amino acid sequences was performed using MUSCLE (https://www.ebi.ac.uk/Tools/msa/muscle/; Edgar, 2004) the multiple sequence alignment *clitool* library. Phylogenetic inference based on the multiply aligned KRTs was conducted through phylogenetic tree construction, by employing neighbor-joining (NJ) and maximum-likelihood (ML) methods. The robustness of the inferred phylogenetic trees was evaluated by bootstrapping (100 bootstrap pseudo-replicates).

NJ trees were constructed using the *Bio.Phylo.TreeConstruction* module in Python. The module *Phylo* from Bio (Cock et al., 2009) was used to obtain the Newick and PhyloXML trees. The PhyloXML format was selected for storing the style of the phylogenetic trees. NJ phylogenetic trees were visualized using Matplotlib (Hunter, 2007), whereas ML trees were visualized with iTOL (Letunic & Bork, 2021). The ML trees were generated using the software package MEGA version 10.1 (Kumar et al., 2018).

### Differential gene expression patterns

RNA sequencing (RNA-Seq) gene expression data for tumor and corresponding normal tissue samples from the TCGA and GTEx databases, respectively, were downloaded from the GEPIA2 (Gene Expression Profiling Interactive Analysis) online web server (http://gepia2.cancer-pku.cn/; Li et al., 2021).

The differentially expressed *KRT* genes between tumor and normal samples were identified using one-way analysis of variance (ANOVA), by setting the cut-off value for absolute log2 fold change ∣log2FC∣ ≥ 2 and FDR-adjusted *p*-value ≤ 0.05. The *KRT* genes and their corresponding values were stored as a server-less and self-contained database using Python SQLite. In order to visualize the differentially expressed genes (DEGs) between tumor and corresponding normal tissue samples, an R script was implemented. The interactions between the different types of cancers and the DEGs were used to generate a network which was manipulated and visualized by Cytoscape (http://www.cytoscape.org/; Shannon et al., 2003).

### Protein-protein interactions (PPI)

A functional network of the interactions among the protein products of the identified cancer-relevant differentially expressed *KRT* genes was generated by utilizing STRING (Search Tool for the Retrieval of Interacting Genes) version 11.0b (Szklarczyk et al., 2021); a database of experimental and predicted, direct (physical) or indirect (functional), associations among genes/proteins, derived from different sources such as high-throughput experiments, biomedical text mining, co-expression or gene fusion. The gene/protein associations detected in STRING were provided as input to Cytoscape (Shannon et al., 2003) for network construction and visualization.

### Natural language processing

Semantic relationships between keratins and cancer for each member of the type I *KRT* gene family were discovered through biomedical literature mining, with the application of Natural Language Processing (NLP) methods (Lauriola, Lavelli & Aiolli, 2022). The scientific literature database MEDLINE/PubMed (https://pubmed.ncbi.nlm.nih.gov/) was searched thoroughly with artificial intelligence techniques using “keratin” AND “cancer” keywords to obtain relevant articles. A Python script was implemented to convert the raw text to proper data structure for further processing. Of the 4,950 candidate articles, 1,377 met the inclusion criteria, namely: (a) written in English, (b) including an abstract, (c) containing adequate text for processing. Finally, the Python regular expression library was utilized to distribute each type of keratin into different groups. Only those keratins with an adequate quantity of relevant articles were considered. The freely accessible Python libraries, natural language toolkit (NLTK: https://www.nltk.org/) and spaCy (https://spacy.io/), were used for text processing including tokenization, parsing, lemmatizing and stemming. The Python scispaCy (https://allenai.github.io/scispacy/) package, containing the spaCy “en_core_sci_lg” model, was used for processing biomedical scientific text; scispaCy’s parsing tools were utilized to retrieve phrases related with the entities in cancer and keratins.

#### Word embeddings

Word2Vec is a NLP system that uses neural networks in order to create a distributed word representations in a *corpus* (Sivakumar et al., 2020). Word2Vec embeddings module was implemented in the Python library Gensim (https://pypi.org/project/gensim/) to train word vectors of the pre-processed text. A list of all word-to-word distances was extracted. To compute the similarity distances between each pair of terms, the *Word2Vec.most_similar* function in the gensim Word2Vec model was used. A continuous bag-of-words (CBOW) algorithm was used, which forms a new text vector representation for predicting other words in the sentence (Liu, 2018). Only those words that appeared more than five times (*i.e*., minimum frequency threshold) were vectorized; iteration was set at 30 (epochs). Increased number of iterations enhanced the performance of Word2Vec, since the algorithm re-learned the relation between words.

#### Data visualization

A list of all term (word)-to-term (word) distances was retrieved. To compute the similarity distances between associated *KRT* genes and related words, the *Word2Vec.most_similar* function in the Gensim Word2Vec model was applied. The highest ranking 50 detected entries were included. Entries with word frequency below 10 were excluded. The interactions between the *KRT* genes and their top 50 similar words (frequency threshold set to 10) were used to generate networks which were manipulated and visualized with Cytoscape (Shannon et al., 2003). Also, for this study, Python SQLite, a server-less and self-contained database was created to easily access, manage, and update the collection of the cancer datasets (https://www.sqlite.org/).

## Results

### Phylogenetic analysis of keratins

**Figure 1:**
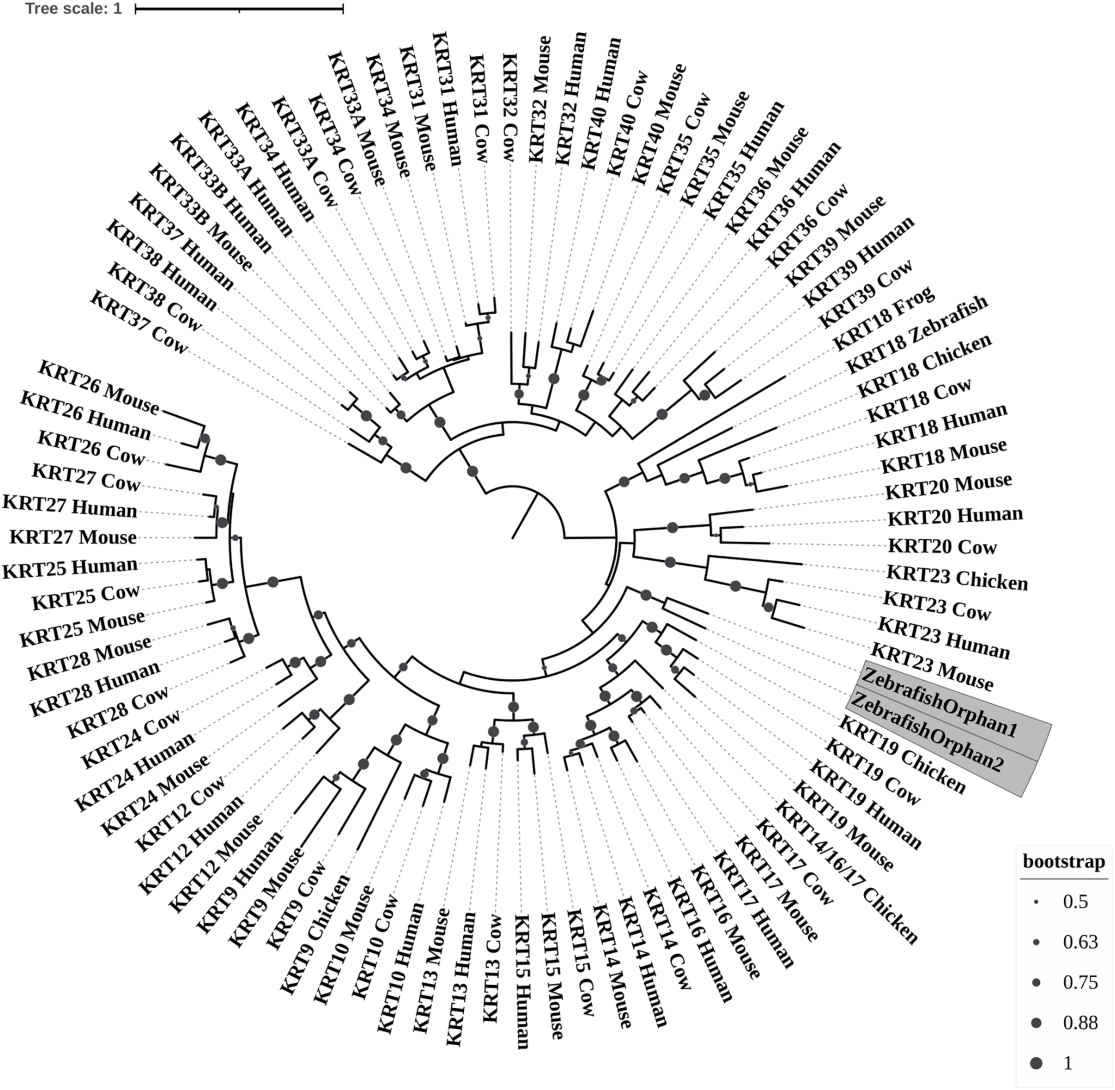
ML radial cladogram of KRT type I protein sequences. The sequences are represented by the species name and the KRT names.

**Figure 2:**
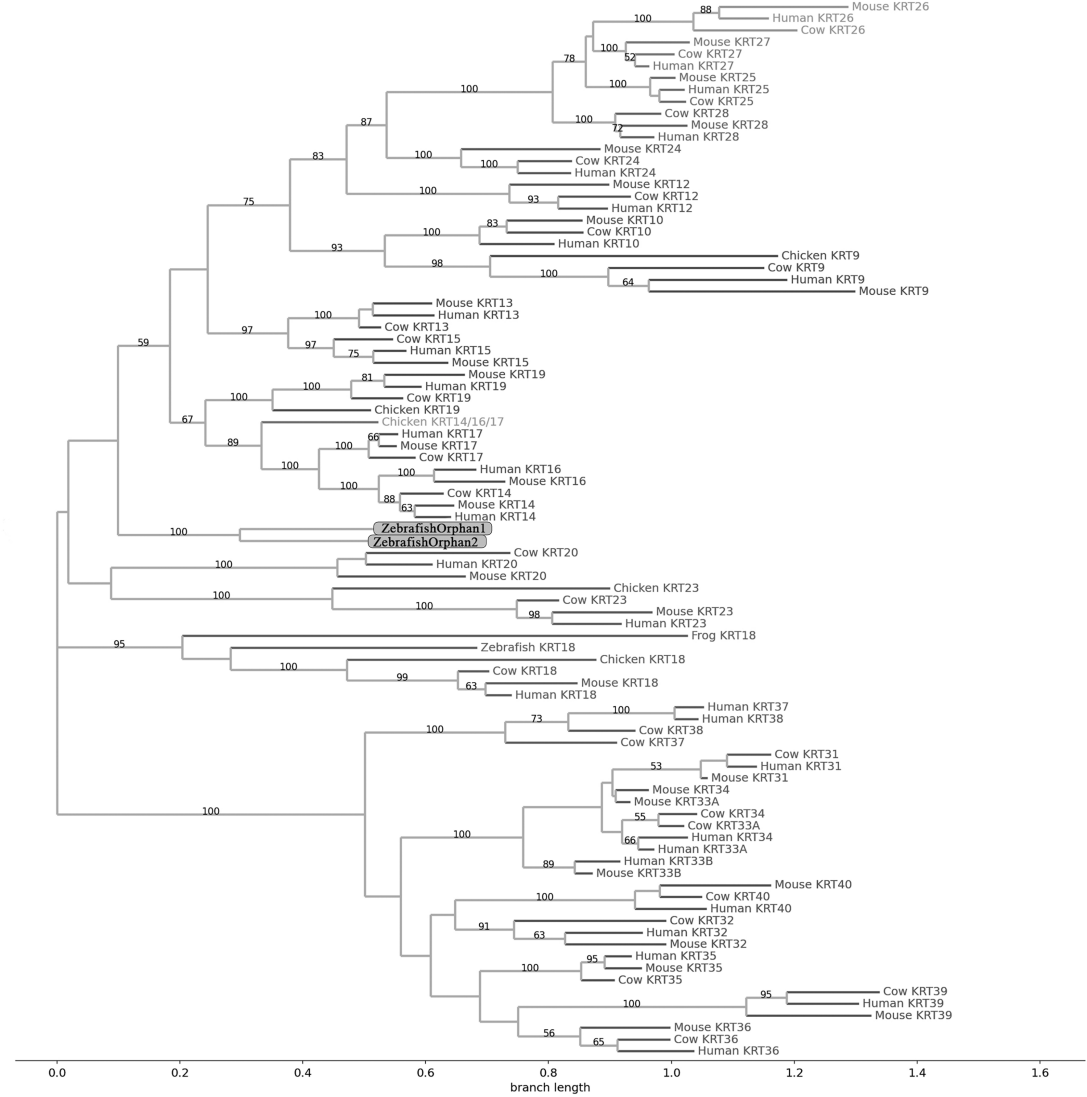
NJ phylogram of KRT type I proteins. Bootstrap values (>50) are shown at the nodes. The branch length at the bottom indicates the length of amino acid substitutions per position.

**Figure 3:**
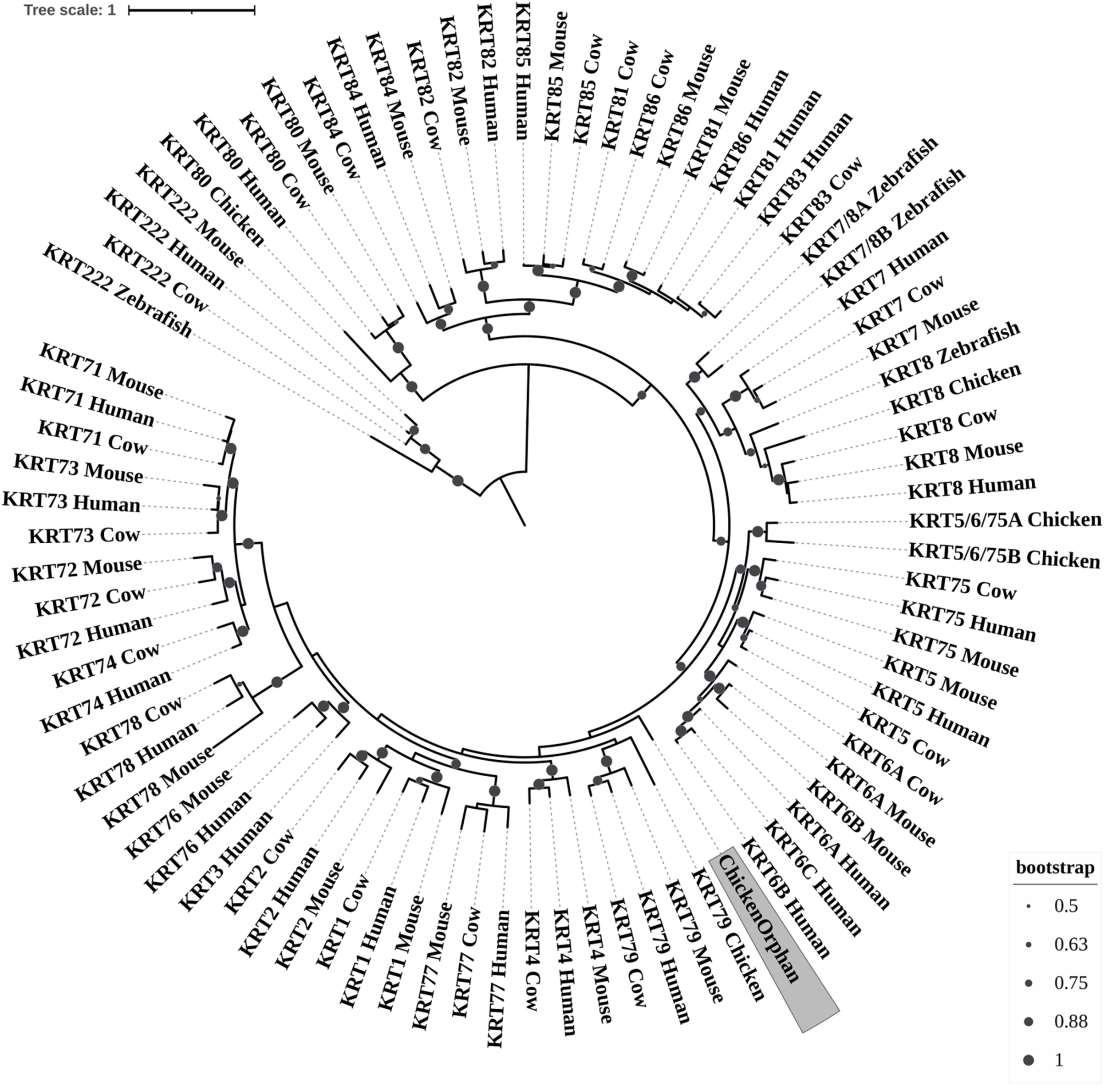
ML radial cladogram of KRT type II protein sequences. The sequences are represented by the species name and the KRT protein names.

**Figure 4:**
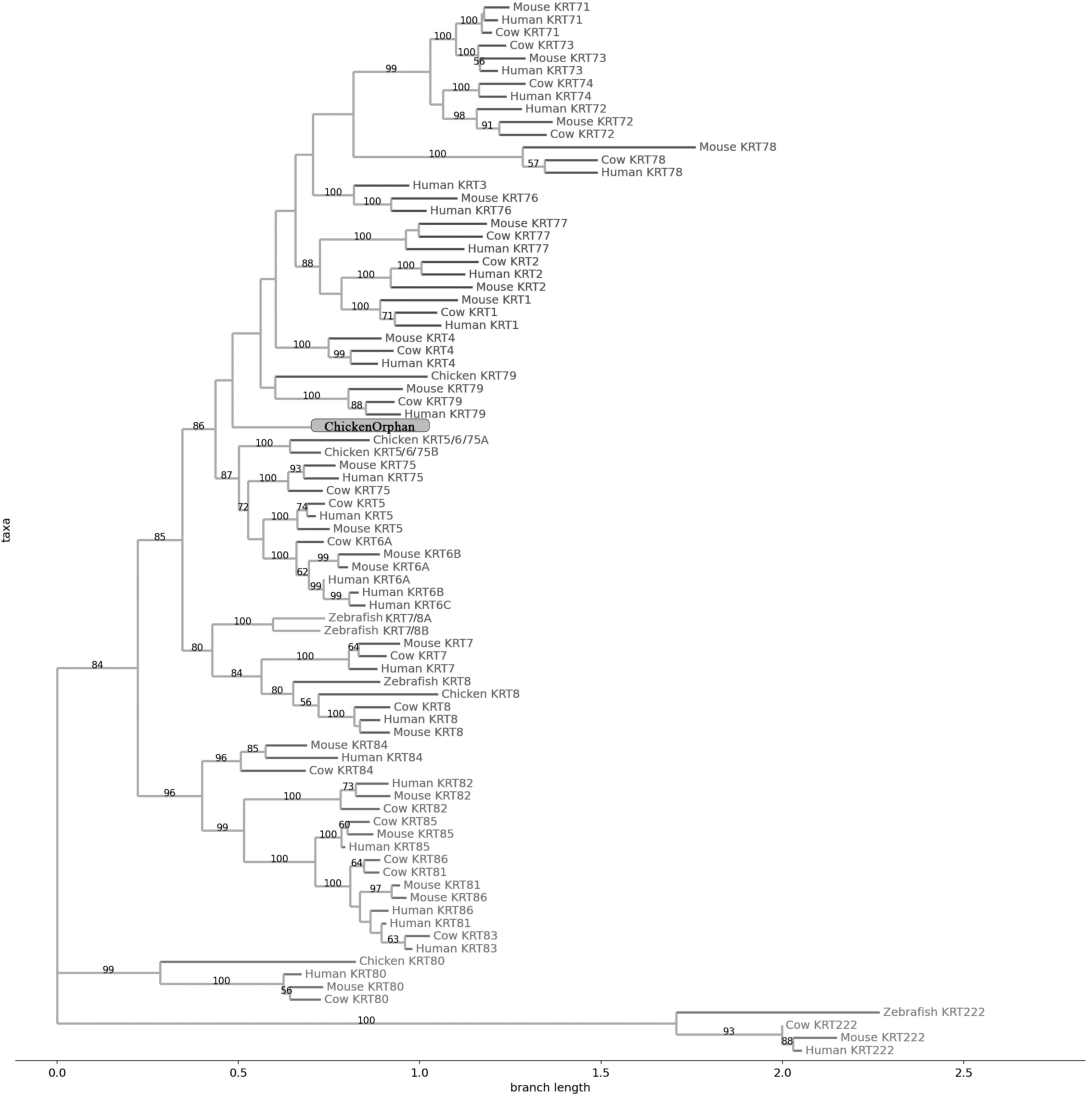
NJ phylogram of KRT type II proteins. Bootstrap values (>50) are shown at the nodes. The branch length at the bottom indicates the length of amino acid substitutions per position.

In order to infer evolutionary relationships among keratins, phylogenetic trees of KRT protein sequences were generated using both the NJ and ML methods. Several major distinct clades were identified in all trees (Figs. 1–4).

### Keratins type I

The two phylogenetic trees (Figs. 1 and 2), reconstructed based on KRT type I proteins, using both methods, are congruent with similar topologies. The zebrafish orphan KRTs form their own distinct clade supported by high bootstrap values, a fact that implies that these are likely the members of the *KRT* family that diverged earliest (‘‘proto-KRTs’’) before the emergence of Amphibia 330 million years ago. One plausible explanation is that a *proto-KRT* gene emerged in an ancestor of Teleostei and a series of lineage-specific gene duplication events gave rise to *KRTorphan1* and *KRTorphan2* found in the contemporary zebrafish genomes.

The primordial gene of the *KRT* family appears to be *KRT18*, since it was detected in zebrafish and frog, leading to the suggestion that it first emerged in an ancestor of Euteleostomi. Interestingly, the branches of the KRT18 clade are exceptionally long, suggesting that the KRT18 members evolved independently and more rapidly compared to the other KRTs.

The largest clade of the reconstructed trees (Figs. 1 and 2) comprises KRT31, KRT32, KRT33, KRT34, KRT35, KRT36, KRT37, KRT38, KRT39 and KRT40 which were identified exclusively in Eutheria, *i.e*., placental mammals. The subclade consisting of KRT37 and KRT38 is basal to the major clade in both trees (Figs. 1 and 2). This result suggests that their corresponding genes were probably the first to emerge through a recent duplication event that took place in primates, since the human KRT37 and KRT38 sequences appear to have strong similarity. Of note, KRT37 and KRT38 murine orthologs were not detected, a fact that implies that the corresponding genes either got lost during evolution or were highly corrupted in glires. The phylogenies indicate that a series of nine mammalian-specific tandem duplication events took place before the Laurasiatheria–Euarchontoglires divergence, thereby yielding those ten paralogs.

Another set of sister clade is formed by KRT20 and KRT23, where KRT23 appears to be more ancient. KRT23 was first detected in chicken, and thus, it is of Amniota origin, while KRT20 was detected exclusively in Eutheria. *KRT20* appears to be the product of a *KRT23* duplication which occurred in a eutherian ancestor presumably after the Mammalia-Sauropsida split.

Additionally, an ancestral KRT14/16/17 was identified in chicken. A *KRT14/16/17* primordial gene of Amniota origin, through a series of mammalian-specific duplication events, presumably has given rise to the three separate *KRT14*, *KRT16* and *KRT17* orthologs found in the mammalian genomes. *KRT14* and *KRT17* appear to have emerged first during the course of mammalian evolution, while *KRT16* arose later, after the Laurasiatheria–Euarchontoglires split, since it was not detected in the Laurasiatheria.

A sister group to the KRT14/16/17 group consists of the KRT19 homologs. The two groups have strong similarity, as confirmed by relatively high bootstrap values (Figs. 1 and 2). KRT19 was also first identified in chicken, and thus, most likely, it emerged in an ancestor of Amniota. Those findings trigger the speculation that *KRT19* and *KRT14/16/17* occurred due to a duplication event which took place in an ancestor of Amniota before the divergence of Mammalia and Sauropsida.

KRT13 and KRT15 together constitute a sister group to the KRT14/16/17–KRT19 group. Both KRT13 and KRT15 are detected exclusively in Eutheria, hence they might have emerged after a series of species-specific duplication events that yielded those mammalian paralogs in an ancestor of Eutheria after the Laurasiatheria-Euarchontoglires split.

Another KRT member that was first detected in Amniota is KRT9, which most likely appeared earlier in amniotic evolution, relatively to KRT14/16/17 and KRT19, since it does not share a high degree of sequence similarity with its other two paralogs. Moreover, as suggested by the topology and the high bootstrap values, KRT9 appears to have strong similarity with KRT10 (Figs. 1 and 2), the latter of which is found only in Eutheria, triggering the speculation that *KRT10* emerged through a mammalian-specific duplication event of *KRT9* that occured most likely after the divergence of Mammalia and Sauropsida.

Finally, KRT12, KRT24, KRT25, KRT26, KRT27 and KRT28 form a separate, highly-supported clade, restricted to Mammalia (Figs. 1 and 2). *KRT12* is most likely the first of this group to arise during the course of mammalian evolution, while *KRT24*, *KRT28*, *KRT25*, *KRT27* and *KRT26* appeared later in the *KRT* family. The phylogenies suggest that a series of four mammalian-specific tandem gene duplication events might have given rise to the corresponding orthologs, apparently after the Laurasiatheria–Euarchontoglires divergence.

### Keratins type II

The two phylogenetic trees (Figs. 3 and 4), reconstructed based on KRT Type II proteins, using both methods, are congruent with similar topologies. On the basis of the reconstructed phylogenies, the primordial gene of the *KRT* family appears to be *KRT222* (‘‘proto-KRTs’’) since it was detected in zebrafish, leading to the suggestion that it first emerged in an ancestor of Teleostei. The branches of KRT222 form their own distinct clade which arises from the basal node with high bootstrap values, indicating that the corresponding gene diverged earliest. Additionally, the KRT222 branch is exceptionally long, suggesting that the KRT222 members evolved independently and more rapidly compared to the other KRTs, and thus, they are distantly related to the rest of the members of the *KRT* family. Therefore, it is intriguing to speculate that the *KRT222* orthologs in the contemporary genomes are probably the products of a series of duplications of an ancestral *KRT222* gene in Teleostei.

Moreover, a distinct clade comprised of the *KRT80* gene products is distantly related to the other KRTs in the trees. KRT80 was first detected in chicken, and thus, it is of Amniota origin, suggesting that an ancestral *KRT80* gene in Amniota gave rise to the *KRT80* orthologs in placental mammals, which evolved more rapidly compared to the fellow Type II keratins.

Another highly-supported distinct large clade of the trees comprises of the *KRT81*, *KRT82*, *KRT83*, *KRT84*, *KRT85* and *KRT86* gene products which are restricted to Eutheria. The subclade consisting of KRT84 is basal to the major clade in both trees (Figs. 3 and 4), suggesting that the *KRT84* gene probably arose first through a recent duplication event that took place in a eutherian ancestor of the contemporary placental mammals. The next gene to emerge during the course of mammalian evolution, according to the phylogenies, appears to be *KRT82* followed by *KRT85*. The genes *KRT86*, *KRT81* and *KRT83* seem to have emerged later since their products’ sequences appear to have strong similarity. Conclusively, the reconstructed phylogenies lead to the speculation that a series of five eutherian-specific tandem gene duplication events might have given rise to those six paralogs.

Also, a set of sister clades is formed by the *KRT7* and *KRT8* gene products. KRT8 appears to be the older of the two, since it was first detected in zebrafish, and thus, it is of Teleostei origin, while KRT7 was identified exclusively in Eutheria. *KRT7* gene appears to be the product of a *KRT8* gene duplication which occurred in a eutherian ancestor presumably after the divergence of Mammalia and Sauropsida, since KRT7 was not detected in chicken.

A clade basal to the KRT7/8 group contains the zebrafish KRT7/8A and KRT7/8B; these two sequences exhibit a strong similarity and they form a discernible highly-supported branch in both trees, suggesting products of a teleost-specific gene duplication event. One plausible explanation is that a *proto-KRT7/8* gene emerged in an ancestor of Teleostei and a series of lineage-specific gene duplication events gave rise to the two paralogous genes found in the contemporary zebrafish genomes. However, those two sequences might have evolved rapidly and separately, since they do not have great similarity with their homologs detected in other species.

In addition, KRT5, KRT6 and KRT75 constitute a distinct group in both trees. KRT6B was identified in mouse for the first time, leading to the suggestion that a gene duplication event, which took place in an early eutherian mammal after the Laurasiatheria – Euarchontoglires divergence, yielded those paralogs. Finally, KRT6C was detected exclusively in human, which triggers the speculation that it emerged through a recent duplication event that took place in primates, since the human KRT6A, KRT6B and KRT6C sequences appear to have a high degree of similarity, as confirmed by high bootstrap values (Figs. 3 and 4). KRT5 members are more similar to KRT6s, suggesting products of the duplication of a eutherian *KRT5/6* gene. Moreover, the KRT75 clade, which comprises a sister group to KRT5/6, appears to have emerged later in the evolution and includes only eutherian KRT members. Two chicken KRT5/6/75 paralogs, which sort into a distinct, well-supported clade, suggest that an ancestral amniotic *KRT5/6/75* gene has given rise to the KRT5, KRT6 and KRT75 members.

An orphan KRT sequence was detected in *Gallus gallus* (Chicken KRTorphan), which bears strong similarity to the chicken paralogs KRT6/75, based on the short branch lengths connecting them, suggestive of short evolutionary distance. Moreover, KRT4 sequences form a discernible clade which is a sister group to KRT79, the latter of which was first detected in chicken, and thus, it is of Amniota origin.

Another distinct clade is comprised of the *KRT1*, *KRT2* and *KRT77* gene products which are restricted to Eutheria. The subclade consisting of KRT77 is basal to the major clade in both trees (Figs. 3 and 4), suggesting that the *KRT77* gene was probably the first to emerge through a duplication event that occurred in a Eutherian ancestor of the contemporary placental mammals. Another lineage-specific gene duplication seems to have yielded the *KRT1* and *KRT2* paralogs.

One distinct clade is formed by the *KRT76* and *KRT3* gene products, which have strong similarity, as confirmed by relatively high bootstrap values (Figs. 3 and 4). Notably, KRT76 was first identified in mouse, suggesting that the *KRT76* gene first arose in an early eutherian mammal, most likely after the divergence of Laurasiatheria and Euarchontoglires. KRT3, on the other hand, was detected exclusively in human, suggesting that it emerged through a recent duplication event that occurred in primates.

The final highly-supported distinct clade of both trees contains the *KRT78*, *KRT71*, *KRT72*, *KRT73* and *KRT74* gene products, which were identified exclusively in Eutheria. The subclade consisting of KRT78 sequences is basal to the major clade in both trees (Figs. 3 and 4), suggesting that the *KRT78* gene was probably the first to emerge in the course of mammalian evolution through duplications that took place in a eutherian ancestor of the contemporary placental mammals. Two sister groups are comprised of the KRT72 and KRT74, and KRT73 and KRT71 sequences, respectively. The two groups appear to share a high degree of similarity, as confirmed by relatively high support values, thus triggering the speculation that a series of three mammalian-specific gene duplication events yielded the *KRT71*, *KRT72*, *KRT73* and *KRT74* paralogs.

### Differential *KRT* gene expression across cancers

**Figure 5:**
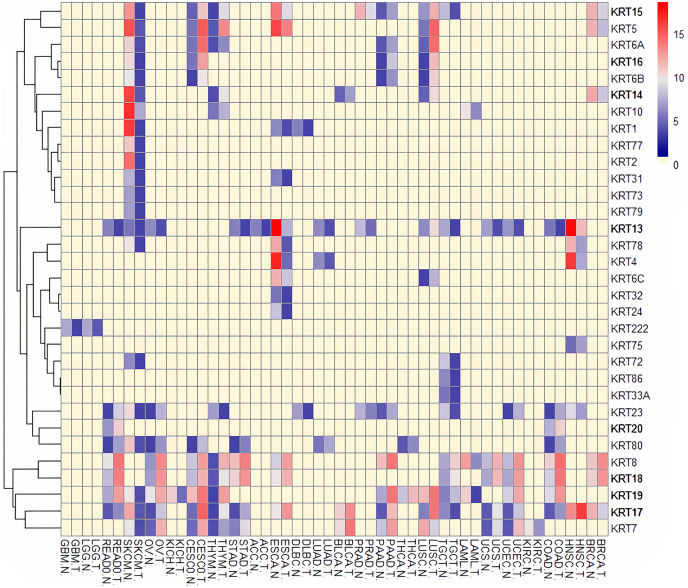
Heatmap of differentially expressed *KRT* genes based on data derived from the cancer SQLite database. Red: up-regulated, Blue: down-regulated, Yellow: no data. T, tumor; N, matched normal.

**Figure 6:**
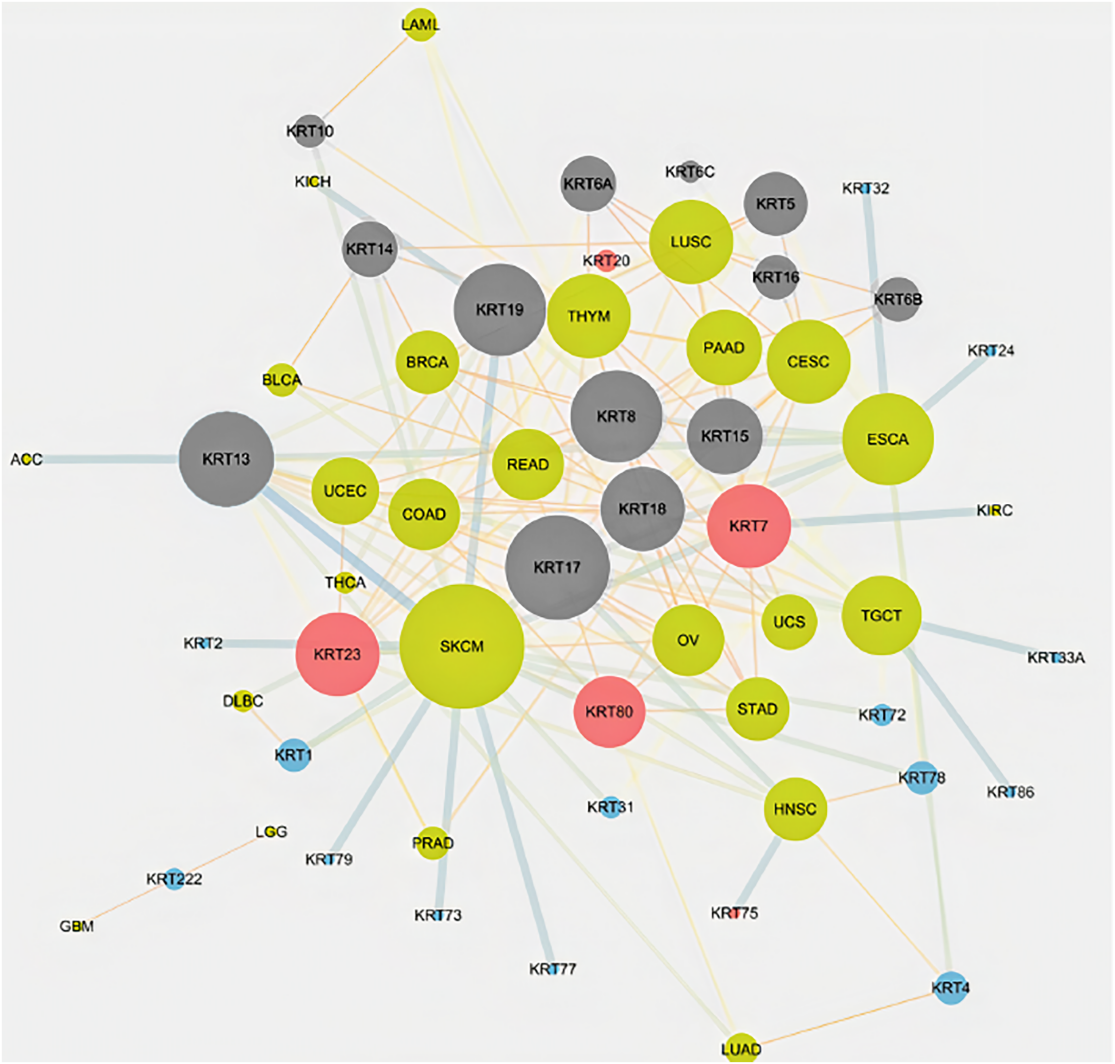
Keratins are linked to multiple cancers. The node size is proportional to the number of direct links. Red and blue color denotes consistent up-regulation and down-regulation across cancers, respectively; gray color indicates, both, up- and down-regulation in the different types of cancers in Table 1.

**Figure 7:**
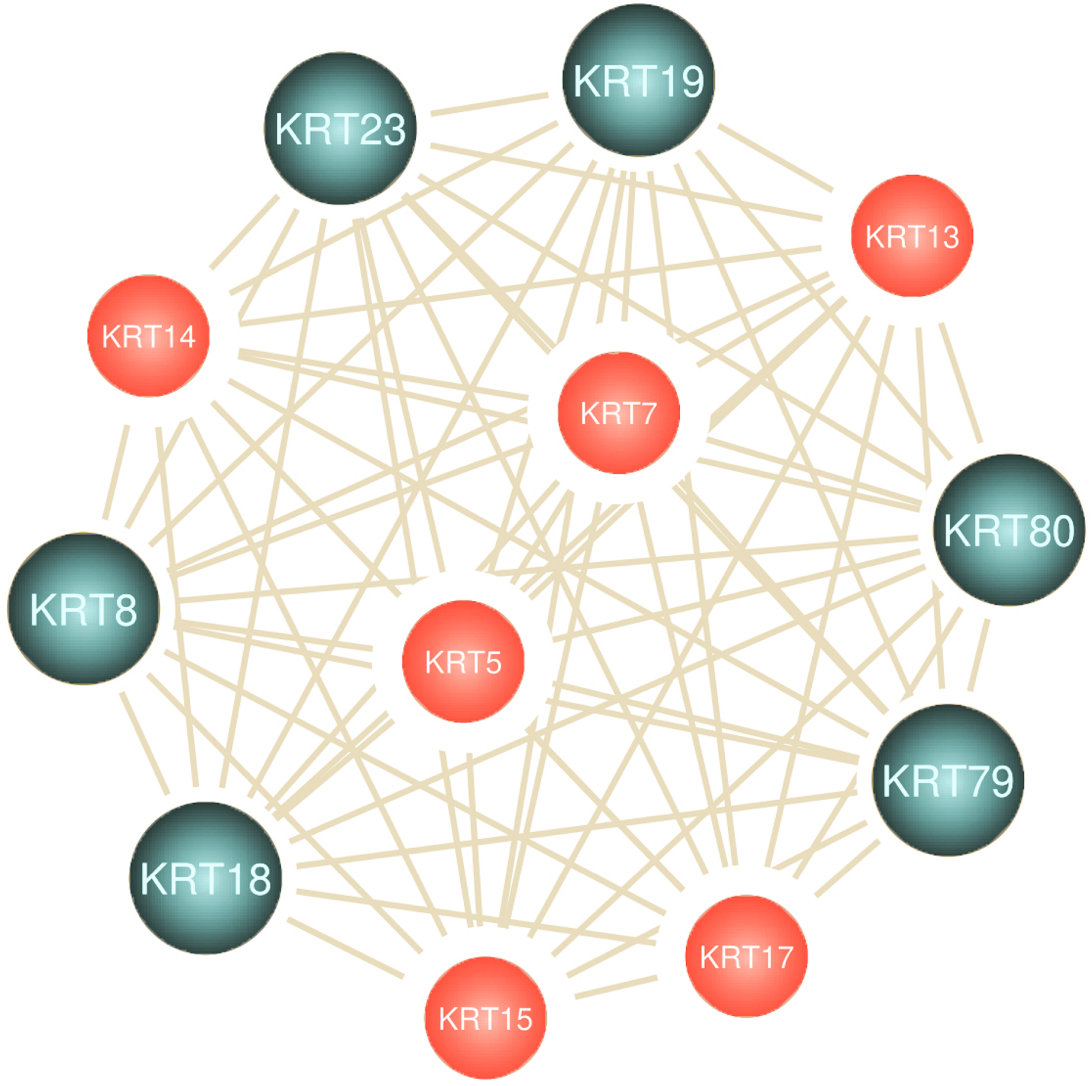
Keratins are linked to multiple cancers. Node size represents the number of direct links.

To assess the role of keratins in cancer, the differential *KRT* gene expression patterns were investigated across cancers. Several *KRT* genes, including the phylogenetically older *KRT8*, *KRT18*, *KRT19*, *KRT23*, *KRT79*, *KRT80* and *KRT222*, were found to be differentially expressed in diverse types of cancers (Table 1 and Fig. 5). Of note, *KRT222*, which was first detected in *Gallus gallus*, was down-regulated specifically in brain neoplasms (lower grade glioma and glioblastoma multiforme). Moreover, the phylogenetically conserved *KRT5*, *KRT7*, *KRT13*, *KRT14*, *KRT15* and *KRT17*, were differentially expressed in multiple types of cancers (Fig. 5, bold). To visualize the relationships between KRTs and cancers, a bipartite network was constructed displaying TCGA-derived cancer-KRT associations by using information from the cancer SQLite database developed in this study. The size of the nodes is proportional to their degree of connectivity. The network is highly interconnected, suggesting associations among diverse cancer types and both types of *KRT* genes (Fig. 6). Of note, the protein products of those *KRTs* differentially expressed in multiple cancers, including the older ones, form a highly interconnected network, suggesting functional or physical associatiosn among them (Fig. 7).

**Table 1:** *KRT* genes, cancer types and gene expression status.

GeneName	TCGA cancer type	Status
KRT1	DLBC, ESCA, SKCM	Down
KRT10	SKCM, LAML	Down
	THYM	Up
KRT13	LUSC	Up
	UCEC, UCS, LUAD, ESCA, HNSC, COAD, ACC, SKCM, STAD, TGCT, READ, PRAD, OV	Down
KRT14	SKCM, BRCA	Down
	BLCA, LUSC, THYM	Up
KRT15	THYM, LUSC, CESC, PAAD	Up
	BRCA, SKCM, TGCT, ESCA, PRAD	Down
KRT16	SKCM	Down
	PAAD, CESC, LUSC	Up
KRT17	LUSC, HNSC, ESCA, COAD, UCEC, BLCA, CESC, STAD, THYM, UCS, PAAD, OV, READ	Up
	SKCM, TGCT, BRCA	Down
KRT18	UCS, THYM, UCEC, READ, OV, TGCT, STAD, CESC, BRCA, COAD, ESCA	Up
KRT19	SKCM, LAML, KICH	Down
	CESC, LUSC, COAD, THCA, TGCT, OV, PAAD, READ, THYM, UCEC	Up
KRT2	SKCM	Down
KRT20	READ, COAD	Up
KRT222	LGG, GBM	Down
KRT23	HNSC, DLBC, SKCM, THYM, TGCT, PRAD	Down
	COAD, READ, PAAD, OV, UCEC	Up
KRT24	ESCA	Down
KRT31	ESCA, SKCM	Down
KRT32	ESCA	Down
KRT33A	TGCT	Down
KRT4	ESCA, HNSC, LUAD	Down
KRT5	LUSC, THYM, CESC	Up
	BRCA, SKCM, ESCA	Down
KRT6A	SKCM	Down
	THYM, PAAD, CESC, LUSC	Up
KRT6B	LUSC, CESC, PAAD	Up
	SKCM	Down
KRT6C	LUSC	Up
	ESCA	Down
KRT7	KIRC, LUSC, SKCM	Down
	BLCA, STAD, THYM, PAAD, OV, CESC, UCS, UCEC	Up
KRT72	SKCM, TGCT	Down
KRT73	SKCM	Down
KRT75	HNSC	Up
KRT77	SKCM	Down
KRT78	SKCM, HNSC	Down
KRT78	ESCA	Down
KRT79	SKCM	Down
KRT8	TGCT, STAD, THYM, READ, OV, PAAD, UCS BRCA, CESC, COAD, ESCA, UCEC	Up
	LAML	Down
KRT80	COAD, CESC, LUAD, OV, READ, STAD, THCA	Up
	SKCM	Down
KRT86	TGCT	Down

**Note:**

ACC, Adrenocortical carcinoma; BLCA, Bladder Urothelial Carcinoma; BRCA, Breast invasive carcinoma; CESC, Cervical squamous cell carcinoma and endocervical adenocarcinoma; COAD, Colon adenocarcinoma; DLBC, Lymphoid Neoplasm Diffuse Large B-cell Lymphoma; ESCA, Esophageal carcinoma; GBM, Glioblastoma multiforme; HNSC, Head and Neck squamous cell carcinoma; KICH, Kidney Chromophobe; KIRC, Kidney renal clear cell carcinoma; LAML, Acute Myeloid Leukemia; LGG, Brain Lower Grade Glioma; LUAD, Lung adenocarcinoma; LUSC, Lung squamous cell carcinoma; OV, Ovarian serous cystadenocarcinoma; PAAD, Pancreatic adenocarcinoma; PRAD, Prostate adenocarcinoma; READ, Rectum adenocarcinoma; SKCM, Skin Cutaneous Melanoma; STAD, Stomach adenocarcinoma; TGCT, Testicular Germ Cell Tumors; THCA, Thyroid carcinoma; THYM, Thymoma; UCEC, Uterine *Corpus* Endometrial Carcinoma; UCS, Uterine Carcinosarcoma.

The aforementioned findings further validate the interrelatedness among keratins and their important implication in multiple cancer pathways.

### KRT and cancer semantic relations

**Figure 8:**
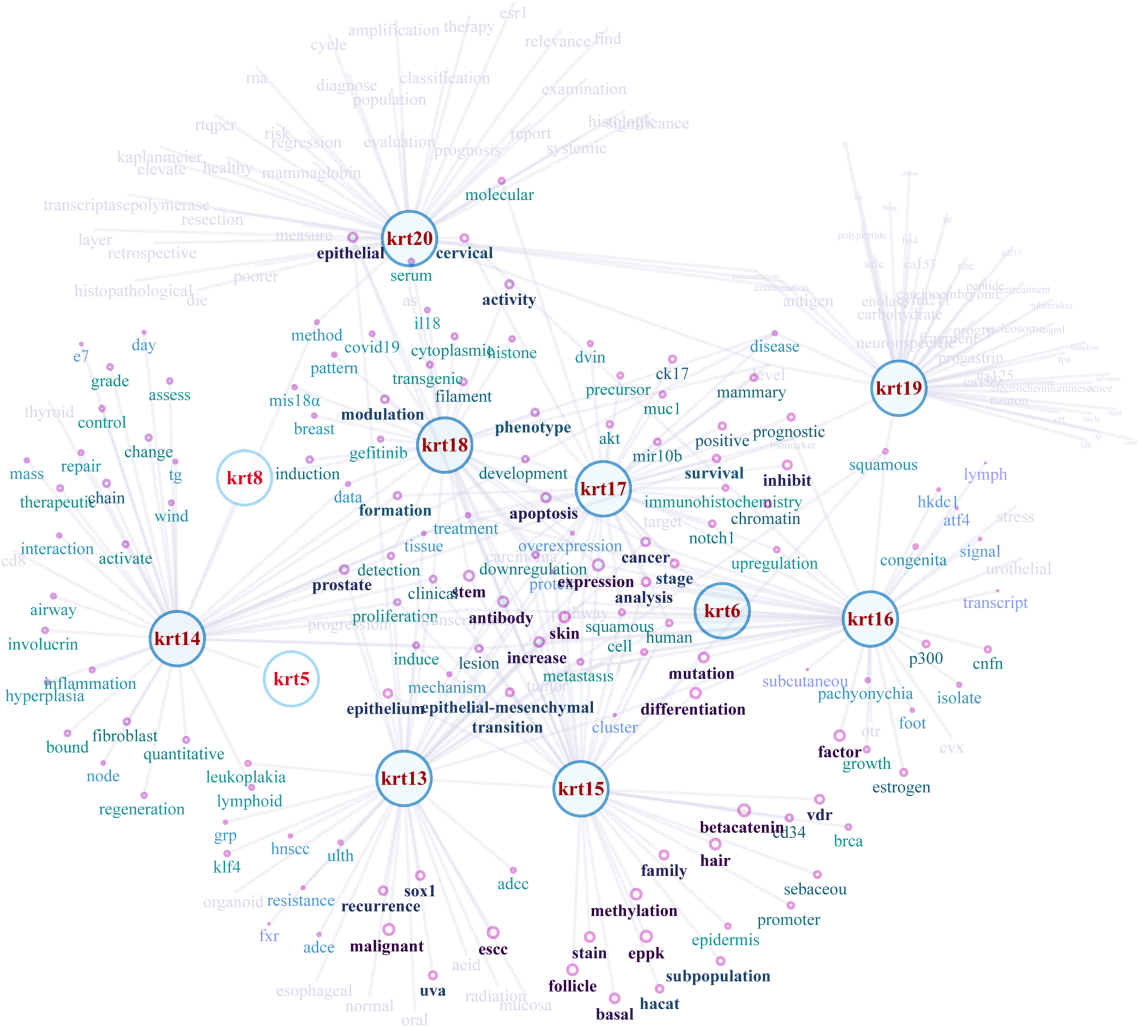
Network depicting the semantic relationships of the top 50 most similar terms to KRTs.

NLP-aided literature mining was performed in order to identify important relationships between type I keratins and cancers. This method allowed the systematic, extensive and comprehensive scrutinization of a vast number of scientific articles for extracting relevant information. Networks illustrating the semantic relations of the highest ranking 50 most similar terms (words) to KRT13, KRT14, KRT15, KRT16, KRT17, KRT18, KRT19 and KRT20 were created (Fig. 8). The nodes represent the words, and the edges denote the semantic associations to selected KRTs with adequate texts in their document. The semantic relations of the top ranking 50 terms (words) were constructed using the similarity distances between each word; a KRT association network was generated which was manipulated and visualized by Cytoscape (http://www.cytoscape.org/; (Shannon et al., 2003)). The word nodes on the network were placed according to their similarity with each KRT.

## Discussion

The reconstructed phylogenies of keratins allowed us to get a glimpse of the evolutionary history of this extended gene family, and gain an understanding of how members of this family are associated with certain pathophysiological processes. According to the results of the phylogenetic analysis, duplication and mutation events during the course of evolution gave rise to the *KRT* gene family, essential for developing novel genes; some *KRT* genes were probably converted to pseudogenes and *vice versa*.

Several members of the *KRT* gene family, including the evolutionarily older *KRT8*, *KRT18*, *KRT19*, *KRT23*, *KRT79*, *KRT80* and *KRT222*, were shown to be differentially regulated in diverse cancer types (Table 1). The expression status of the *KRT* genes under study varies in different types of cancers, *i.e*., some are up-regulated, and, conversely, some are down-regulated in different cancer types. Hence, at least in cancer-bearing species, the evolutionarily conserved genes have likely resist evolutionary changes, so as to carry out critical functions in carcinogenesis, consistent with the hypothesis postulated by Makashov, Malov & Kozlov (2019) that oncogenes and tumor suppressor genes represent the evolutionarily oldest classes of genes in eukaryotic species. Furthermore, the Amniota-specific *KRT8*, *KRT19*, *KRT23* and *KRT80* were shown to be up-regulated in ovarian cancer. Given that chicken is currently the only animal model available to investigate the etiology and progression of human ovarian cancer (Hawkridge, 2014), the respective orthologs could be taken into consideration in the study of ovarian cancer. Moreover, the *KRT18* ortholog first appeared in *Danio rerio*. Notably, *KRT18* was under-expressed in skin cutaneous melanoma relative to normal tissue (Table 1). Since zebrafish represents a unique experimental model organism for studying melanoma development, progression and treatment (Bootorabi et al., 2017), the teleost *krt18* gene could also be considered in the melanoma clinical research. Furthermore, an amphibian *KRT18* ortholog was detected in frog. According to Hardwick & Philpott (2018), *Xenopus* model systems have diverse applications in cancer research and, particularly, in tumor immunity.

The constructed KRT-oriented networks, using word embedding, provide valuable information on how related words in a given text dataset have semantic and syntactic similarity with a given keratin. Our results support that there is a significant association between keratins and a number of cancer biomarkers (Fig. 8).

Keratin 18 represents a robust diagnostic and prognostic biomarker for human cancers (Menz et al., 2021). For instance, the expression patterns of conventional tumor markers, such as the proliferating cell nuclear antigen (PCNA) and the minichromosome maintenance protein 3 (MCM3) in breast cancer were found to be similar to those of *KRT18*. Besides, *KRT18* was significantly correlated with the loss of estrogen and progesterone receptors (Ha et al., 2011). In the present study, *KRT18* was over-expressed in breast invasive carcinoma (BRCA) (Table 1), further supporting that KRT18 could represent a candidate biomarker in breast cancer for predicting poor prognosis in breast cancer. Moreover, it has been suggested that caspase-cleaved KRT18, a serum apoptosis product, could be a functional biomarker for predicting the response of breast carcinomas to chemotherapy (Olofsson et al., 2007).

It has been demonstrated that the evolutionarily old *KRT19* is differentially expressed in several types of cancers. In the present study, *KRT19* was found overexpressed in breast, colon, lung, liver and thyroid cancer (Table 1), consistently with previous reports; *KRT19* was associated with poor clinical outcomes in cancer patients as well (Kawai et al., 2015; Saha et al., 2019; Wang et al., 2019; Yuan et al., 2021). KRT19 represents one of the factors determining tumor response to chemo/radiotherapy (Bozionellou et al., 2004; Saha et al., 2018).

Low expression of *KRT15* in breast invasive carcinoma (Table 1) was associated with unfavorable prognosis (Zhong et al., 2021). Moreover, KRT15 was demonstrated to promote migration and invasion of colorectal cancer cells partly *via* β-catenin-mediated signaling (Chen & Miao, 2022); β-catenin, which regulates cell-cell adhesions (Brembeck, Rosario & Birchmeier, 2006), was also found to be semantically related with KRT15 (Fig. 8). Furthermore, the phylogenetically (Figs. 1 and 2) and semantically related (Fig. 8) KRT13 and KRT15 are also directly associated in oral cancers (Khanom et al., 2012). *KRT13* is transcriptionally up-regulated by KLF4 to induce differentiation of esophageal squamous cell carcinoma (He et al., 2015). Likewise, *KRT15* is over-expressed in esophageal carcinoma (Cancer Stat Facts: Leukemia—Acute Myeloid Leukemia (AML), https://seer.cancer.gov/statfacts/html/amyl.html). However, *KRT13* and *KRT15* were found to be down-regulated in TCGA-derived esophageal carcinoma samples (Table 1). It has also been shown that *KRT13* is transcriptionally suppressed during TGF-β1-induced EMT (Hatta et al., 2018), suggesting implication of KRT13 in a hallmark of cancer, *i.e*., invasion and metastasis.

Cancer-associated fibroblasts were shown to exert a powerful stimulatory effect on the expression of KRT14, which is a basal/myoepithelial marker (Dvorankova et al., 2012). KRT14 enhances the metastatic potential of lung cancer cells, promotes cell invasion of salivary adenoid cystic carcinoma, and is also correlated with worse patient prognosis (Gao et al., 2017). In addition, keratin 14 is correlated with nodal metastasis and unfavorable prognosis in human lung adenocarcinoma *via Gkn1* induction (Yao et al., 2019). Similarly, in our study, the corresponding *KRT14* transcripts were elevated in lung squamous cell carcinoma (Table 1). Based on the human protein atlas (HPA) (https://www.proteinatlas.org), KRT14 is a favorable prognostic biomarker for breast cancer, consistent with our findings wherein the *KRT14* gene expression is reduced in breast invasive carcinoma (Table 1). Moreover, transcriptional up-regulation of *KRT14*, and down-regulation of *KRT15* and *KRT19* was observed in oral squamous cell carcinoma (OSCC). Also, deregulated *KRT15* and *KRT19* expression was observed in well-differentiated OSCC as compared to moderately/poorly differentiated OSCC (Khanom et al., 2012).

Increased *KRT16* expression is highly associated with weak differentiation, augmentation of lymph node metastasis, worse survival outcome and advanced stages of OSCC. Also, inhibition of KRT16 resulted to reduced OSCC progression and chemoresistance, whereas *KRT16* silencing improved chemosensitivity (Huang et al., 2019). Moreover, there is a significant correlation between enhanced *KRT16* expression and poor overall survival in metastatic breast cancer patients (Joosse et al., 2012). Based on the HPA, keratin 16 is enhanced in cervical cancer and it is a poor prognostic biomarker for pancreatic cancer. Consistent with the latter, in our study, the corresponding *KRT16* gene was found to be significantly up-regulated in pancreatic adenocarcinoma as well as the cervical squamous cell carcinoma and endocervical adenocarcinoma (Table 1).

Keratin 17 has been underscored as an emerging diagnostic, prognostic, and predictive biomarker (Yang, Zhang & Wang, 2019), based on preclinical and clinical cancer studies. According to Baraks et al. (2022), KRT17 is implicated in eight out of ten deadly hallmarks of cancer. KRT17 triggers the AKT-mediated signaling pathway and induces EMT, while it is strongly correlated with malignant transformation and worse prognosis in esophageal squamous cell carcinoma (ESCC) patients. Therefore, KRT17 may serve as a therapeutic target for the treatment of ESCC. Increased level of KRT17 is directly correlated with the progression of pancreatic cancer (Chen et al., 2020). In addition, keratin 17 is considered a novel cytologic biomarker for accurately distinguishing between recurrent urothelial carcinoma and benign urothelial cells (Babu et al., 2021). Moreover, according to HPA results, KRT17 is over-expressed in cervical and head and neck cancers and represents a favorable prognostic marker for breast cancer. In agreement with the aforementioned findings, in our study, *KRT17* is over-expressed in the head and neck squamous cell carcinoma, esophageal carcinoma, bladder urothelial carcinoma, pancreatic adenocarcinoma as well as cervical squamous cell carcinoma and endocervical adenocarcinoma, whilst it is down-regulated in breast invasive carcinoma (Table 1).

Quantitating *KRT20* expression by RT-PCR is a very sensitive and accurate method to detect unknown lymph node metastases. In addition, by measuring *KRT20* mRNA expression in lymph nodes is essential for exact tumor staging and for postoperative adjuvant treatment of colorectal cancer patients (Chen et al., 2004). Studies on pancreatic carcinoma, gastrointestinal cancers, colorectal carcinoma and miscellaneous tumors suggest that it is convenient to detect circulating tumor cells based on KRT20 RT-PCR assays (Joosse et al., 2012; Lukyanchuk et al., 2003). According to Eckstein et al. (2018), high *KRT5* and low *KRT20* expression defines distinct prognostic subgroups in urothelial bladder cancer. Moreover, according to HPA results, keratin 20 is enriched in colorectal cancer. Consistently, in our study, enhanced expression of *KRT20* was found in colon and rectum adenocarcinoma (Table 1).

The findings of the present study highlight the prominent role of keratins in mammalian cancers. Members of the keratin family could serve as robust diagnostic and prognostic cancer biomarkers as well as potential therapeutic targets. Notably, the evolutionary conservation of several *KRT* genes across taxa points to the importance of using non-mammalian model organisms in functional studies towards investigating the etiology, development, progression, and treatment of cancer.

## Conclusions

In this study, cross-disciplinary systems biology methods were employed by combining phylogenetics, biological literature mining, differentially expressed gene profiles, and biological networks to investigate evolutionarily conserved *KRT* genes implicated in different aspects of cancer. The findings of this study could have potential application in the clinical setting, where the phylogenetically preserved genes can be exploited as diagnostic or prognostic tumor markers. Furthermore, the detection of evolutionarily conserved *KRTs* in non-mammalian species highlights the possible importance of using these organisms as model systems in cancer research.

## Supplemental Information

10.7717/peerj.15099/supp-1 Supplemental Information 1 HGNC IDs, approved symbols, names, and synonyms for Type I KRT genes.

10.7717/peerj.15099/supp-2 Supplemental Information 2 HGNC IDs, approved symbols, names, and synonyms for Type II KRT genes.

## Additional Information and Declarations

### Data Availability

The following information was supplied regarding data availability: The approved symbols and names of *Homo sapiens* keratins were collected from the HUGO Gene Nomenclature Committee (HGNC) database (https://www.genenames.org/). The symbols and accession numbers are available in the Supplemental Tables.
