# Supplementary material for: “In the light of evolution:” keratins as exceptional tumor biomarkers
Source: PeerJ. 2023 Mar 17;11:e15099. doi: 10.7717/peerj.15099 (PMC10026720; doi:10.7717/peerj.15099)
Supplement: Supplemental Information 1 [file peerj-11-15099-s001.docx]

**Table S1.** HGNC IDs, approved symbols, names, and synonyms for Type I *KRT* genes.

| HGNC ID (gene) | Approved symbol | Approved name | Previous symbols | Aliases |
| --- | --- | --- | --- | --- |
| 6412 | KRT1 | keratin 1 | EHK1 | KRT1A |
| 6439 | KRT2 | keratin 2 | KRT2A | KRTE |
| 6440 | KRT3 | keratin 3 |  | CK3, K3 |
| 6441 | KRT4 | keratin 4 | CYK4 | CK4, K4 |
| 6442 | KRT5 | keratin 5 | EBS2 | KRT5A |
| 6443 | KRT6A | keratin 6A | KRT6C, KRT6D | CK6C, K6C, CK6D, K6D |
| 6444 | KRT6B | keratin 6B | KRTL1 |  |
| 20406 | KRT6C | keratin 6C | KRT6E |  |
| 6445 | KRT7 | keratin 7 |  | K7, CK7, K2C7, SCL |
| 6446 | KRT8 | keratin 8 |  | CARD2, K8, CK8, CYK8, K2C8, KO |
| 28927 | KRT71 | keratin 71 |  | KRT6IRS, KRT6IRS1, K6IRS1 |
| 28932 | KRT72 | keratin 72 |  | K6IRS2, KRT6IRS2, KRT6, K6irs |
| 28928 | KRT73 | keratin 73 |  | KRT6IRS3, K6IRS3 |
| 28929 | KRT74 | keratin 74 |  | K6IRS4, KRT5C, KRT6IRS4 |
| 24431 | KRT75 | keratin 75 |  | K6HF |
| 24430 | KRT76 | keratin 76 |  | HUMCYT2A, KRT2B, KRT2P |
| 20411 | KRT77 | keratin 77 | KRT1B |  |
| 28926 | KRT78 | keratin 78 |  | K5B |
| 28930 | KRT79 | keratin 79 |  | K6L, KRT6L |
| 27056 | KRT80 | keratin 80 |  | KB20 |
| 6458 | KRT81 | keratin 81 | KRTHB1 | Hb-1 |
| 6459 | KRT82 | keratin 82 | KRTHB2 | Hb-2 |
| 6460 | KRT83 | keratin 83 | KRTHB3 | Hb-3 |
| 6461 | KRT84 | keratin 84 | KRTHB4 | Hb-4 |
| 6462 | KRT85 | keratin 85 | KRTHB5 | Hb-5 |
| 6463 | KRT86 | keratin 86 | KRTHB6 | MNX, Hb6 |
| 28695 | KRT222 | keratin 222 | KRT222P | KA21, MGC45562 |
