# Supplementary material for: “In the light of evolution:” keratins as exceptional tumor biomarkers
Source: PeerJ. 2023 Mar 17;11:e15099. doi: 10.7717/peerj.15099 (PMC10026720; doi:10.7717/peerj.15099)
Supplement: Supplemental Information 2 [file peerj-11-15099-s002.docx]

**Table S2.** HGNC IDs, approved symbols, names, and synonyms for Type II KRT genes.

| HGNC ID (gene) | Approved symbol | Approved name | Previous symbols | Aliases |
| --- | --- | --- | --- | --- |
| 6447 | KRT9 | keratin 9 |  | EPPK, K9, CK-9 |
| 6413 | KRT10 | keratin 10 | KPP | K10, CK10 |
| 6414 | KRT12 | keratin 12 |  | K12 |
| 6415 | KRT13 | keratin 13 |  | K13, CK13, MGC3781, MGC161462 |
| 6416 | KRT14 | keratin 14 | EBS3, EBS4 |  |
| 6421 | KRT15 | keratin 15 |  | K15, CK15, K1CO |
| 6423 | KRT16 | keratin 16 |  | NEPPK |
| 6427 | KRT17 | keratin 17 | PCHC1 |  |
| 6430 | KRT18 | keratin 18 |  |  |
| 6436 | KRT19 | keratin 19 |  | K19, CK19, K1CS, MGC15366 |
| 20412 | KRT20 | keratin 20 |  | CK20, K20, MGC35423 |
| 6438 | KRT23 | keratin 23 |  | K23, DKFZP434G032, HAIK1, CK23, MGC26158 |
| 18527 | KRT24 | keratin 24 |  | FLJ20261, MGC138169, MGC138173 |
| 30839 | KRT25 | keratin 25 | KRT25A |  |
| 30840 | KRT26 | keratin 26 | KRT25B |  |
| 30841 | KRT27 | keratin 27 | KRT25C |  |
| 30842 | KRT28 | keratin 28 | KRT25D |  |
| 6448 | KRT31 | keratin 31 | KRTHA1 | Ha-1 |
| 6449 | KRT32 | keratin 32 | KRTHA2 | Ha-2 |
| 6450 | KRT33A | keratin 33A | KRTHA3A | Ha-3I, Krt1-3 |
| 6451 | KRT33B | keratin 33B | KRTHA3B | Ha-3II |
| 6452 | KRT34 | keratin 34 | KRTHA4 | Ha-4 |
| 6453 | KRT35 | keratin 35 | KRTHA5 | Ha-5 |
| 6454 | KRT36 | keratin 36 | KRTHA6 |  |
| 6455 | KRT37 | keratin 37 | KRTHA7 |  |
| 6456 | KRT38 | keratin 38 | KRTHA8 |  |
| 32971 | KRT39 | keratin 39 |  | KA35 |
| 26707 | KRT40 | keratin 40 |  | FLJ36600, KA36 |
